# Binge eating symptomatology in overweight and obese patients with schizophrenia: a case control study

**DOI:** 10.1186/1744-859X-5-15

**Published:** 2006-09-12

**Authors:** Yasser Khazaal, Emmanuelle Frésard, François Borgeat, Daniele Zullino

**Affiliations:** 1 Department of Psychiatry, University Hospital of Vaud, Echallens 9, 1004 Lausanne, Switzerland; 2 University Hospitals of Geneva, Division of Substance Abuse, Rue Verte 2, 1205 Geneva, Switzerland

## Abstract

**Objective:**

The purpose of this study was to assess whether severe overweight schizophrenic treated patients differ from controls and from pairs in binge eating symptomatology.

**Method:**

Current body mass index (BMI) and the binge eating status were assessed cross- sectionally in 40 schizophrenic outpatients and 40 non-psychiatric controls. In each group half of the subjects were severe overweight (BMI ≥ 28) or obese.

**Results:**

Pearson Chi-square analysis shows a higher number of subjects with binge symptomatology in the group of patients with schizophrenia having BMI ≥ 28 (Pearson Chi-square = 8.67, p = 0.034). Among subjects with BMI ≥ 28, 60% of patients with schizophrenia and 30% of controls have binge eating symptomatology.

**Conclusion:**

This result may odds to the understanding of weight gain associated with antipsychotics and underscores the importance of assessing binge eating behaviour during treatment and prevention of obesity in this population.

## Background

Weight gain (WG) induced by Antipsychotic drugs (AP) occurs in up to 50 % of patients under chronic administration of such medication [1]. The mechanisms of AP causing WG are complex as they also involve serotoninergic, histaminergic, dopaminergic and adrenergic neurotransmissions [1]. The WG is mediated by an increase in global caloric intake, probably due to appetite and/or satiety alterations [2].

Patients frequently reported their problems associated with WG such as reduced self-esteem and repeated unsuccessful dietary trials [3,4] as attempts to counterregulate weight gain arising during AP therapy.

Binge eating disorder (BED) is a provisional new eating disorder diagnosis [5] described in the DSM-IV research criteria. It is characterized as a pattern of recurrent episodes of binge eating in the absence of extreme weight control measures. Those episodes occur (at least twice a weekly over a 6 month period to met the diagnostic criteria as outlined in DSM-IV. This disorder have a prevalence rates of 23–55% in individuals seeking treatment for obesity compared to 2–3% in community samples [6,7]. In the obese population, binge eaters differ from non binge eaters in aspects of eating disorder psychopathology and psychiatric co-morbidity [8,9]. Increased perception of poor body image and lower weight self efficacy are significantly related to BED [7]. Furthermore, binge eaters tend to experience a higher level of emotional distress [10]. Those characteristics argue for an assessment of this eating disorder in patients with schizophrenia, a population more vulnerable to distress and low self esteem. A previous descriptive study found a high prevalence of BED (12,2%) in psychotic patients treated by clozapine or olanzapine, especially in the high body mass index (BMI) subgroup [3].

The purpose of this study is to assess binge eating symptomatology in a group of individuals receiving treatment for schizophrenia and a group of non-psychiatric controls.

The groups were delineated by BMI for a comparison between individuals with high and average weight.

## Methods

### Subjects

The study sample consisted of 40 schizophrenic (DSM-IV) outpatients (19 female, mean age 33.8 ± 9.1) and 40 non-psychiatric controls (21 female, mean age 35.5 ± 10.8). Both groups were each composed of two subgroups: one severely overweight subgroup (defined as a BMI > 28), and a comparison sample (BMI < 28). All patients with schizophrenia were treated by atypical AP. The WG induced by AP drugs is not necessarily associated to obesity. Due to this consideration, the threshold of BMI = 28 was chosen.

The recruitment procedure was as follows: The study was systematically proposed during regular consultation to outpatients with schizophrenia until reaching 20 patients in each of the BMI subgroups. In total, the study was proposed to 41 outpatients over a 6 month period. Only one patient with obesity has refused to participate in the study. Controls consisted of clinic workers, informed through local advertising.

Participants gave informed consent and local Ethical Committee approval was obtained for the study.

### Design and measures

The present survey was established as a cross-sectional case-control study. Subjects' BMI was calculated as weight in kilograms divided by the square of height in meters.

Binge eating status was assessed through a clinical interview, using DSM-IV criteria (SCID-IV) [11]. To be able to correctly identify binge eating episodes and frequency, detailed description of the binge eating behaviour was obtained from each subject by a senior psychiatrist or psychologist. Purging and other compensatory behaviours were also investigated. With regard to this assessment, patients and controls were classified as having (1)no bingeing, (2) binge episodes less than 2 days per week (BS), (3) BED or (4) bulimia nervosa (BN).

Psychiatric status was assessed through a chart review, medical doctor referee and psychiatrist interview.

### Data analyses

Statistical analysis was performed by SPSS 12.0 program.

An initial exploratory analysis involved calculation of means and standard deviation for age, gender and BMI. Differences between the groups were tested using Kruskall-Wallis nonparametric test (for age and BMI) and chi-square tests (for gender).

Group differences in the BED and the BS prevalence were compared using the Pearson Chi-square test.

## Results

The characteristics of the 4 groups are shown in Table 1. Kruskal Wallis test revealed no differences with regard to age (f = 1.2, df = 3, p = 0.16). No significant differences were found with regard to gender distribution (Chi2 = 2.8, df = 3, p = 0.42). As expected by the study design, Kruskal wallis non parametric test, shows a statistically significant BMI difference between groups (p < 0.0001), whereas no difference was shown between high BMI schizophrenic and non schizophrenic groups (p = 0.93).

**Table 1:** Characteristics of the 4 groups N = 80

	Patients with schizophrenia		Non-psychiatric subjects	
	BMI < 28	BMI ≥ 28	BMI < 28	BMI ≥ 28
	N = 20	N = 20	N = 20	N = 20

Age (mean ± sd)	31.7 ± 9.2	36.1 ± 8.9	33.9 ± 13.0	37.2 ± 8.1
BMI (mean ± sd)	23.6 ± 2.2	32.9 ± 6.1	21.1 ± 2.5	33.8 ± 4.9
(N. female)	9	10	13	8

Binge eating status is shown in Table 2. We found that 17/40 of patients with schizophrenia and 10/40 of controls have binge eating symptomatology (BS or BED). Pearson Chi-square analysis shows a higher number of subjects with binge symptomatology in the group of patients with schizophrenia having BMI ≥ 28 (Pearson Chi-square = 8.67, p = 0.034). Among subjects with BMI ≥ 28, 60% of patients with schizophrenia and 30% of controls have binge eating symptomatology.

**Table 2:** Binge status

	Patients with schizophrenia		Non-psychiatric subjects		
	BMI < 28	BMI ≥ 28	BMI < 28	BMI ≥ 28	
BED	N = 2 (10%)	N = 7 (35%)	N = 0 (0%)	N = 2 (10%)	
BS	N = 3 (15%)	N = 5 (25%)	N = 4 (20%)	N = 4 (20%)	
No BED nor BS Observed counts	15/20 (75%)	8/20 (40%)	16/20 (80%)	14/20 (70%)	
BED or BS (binge symptomatology) Observed counts	5/20 (25%)	12/20 (60%)	4/20 (20%)	6/20 (30%)	Chi-square = 8,67; p = 0.034

## Discussion

The results in this study indicate that BED and BS are common behavior among overweight individuals with schizophrenia undergoing antipsychotic drug treatment. This finding confirms previous observations of high BED rates in the high BMI schizophrenic patient subgroup [12]. The principal finding of this study is the significantly higher BED and BS prevalence in the high BMI schizophrenic group than in their relative controls. This phenomenon observed in patients with schizophrenia having BMI ≥ 28 kg/m2, indicating that this symptomatology is not a specific correlate of antipsychotic treatment or schizophrenia but a clinical correlate of high BMI in those patients.

We can hypothesise that in certain triggered conditions (WG induced by antipsychotic drugs), patients with schizophrenia are more likely to develop BED and BS. This phenomena may be due to a higher level of emotional vulnerability, a predisposing factor to both severe overweight, BED and BS [13]. The conjunction of these factors could explain the relatively high level of binge eating symptoms observed in this study.

Due to absence of non schizophrenic psychiatric controls and the correlation nature of this study, we cannot causally link intake of antipsychotic drugs, BED, BS and WG.

Limitations of this study included the small number of controls in regard to the prevalence of BED in non overweight patients; unknown AP drugs adherence; absence of prospective design as well as absence of non schizophrenic psychiatric controls.

Nevertheless, the present results suggest that binge eating symptomatology may play an important role in the initiation and maintenance of the WG phenomenon observed in at least part of patients with schizophrenia. Finally, management of AP induced WG should take into account possible comorbid binge eating symptomatology leading to consider specific therapies such as cognitive and behavioural treatment adapted to those patients.
